# Brain Correlates of Self-Evaluation Deficits in Schizophrenia: A Combined Functional and Structural MRI Study

**DOI:** 10.1371/journal.pone.0138737

**Published:** 2015-09-25

**Authors:** Shuping Tan, Yanli Zhao, Fengmei Fan, Yizhuang Zou, Zhen Jin, Yawei Zen, Xiaolin Zhu, Fude Yang, Yunlong Tan, Dongfeng Zhou

**Affiliations:** 1 Institute of Mental Health, Peking University, Beijing, 100191, China; 2 Center of Psychiatry Research, Beijing Huilongguan Hospital, Beijing, 100096, China; 3 Magnetic Research Imaging Unit, The 306th Hospital of PLA, Beijing, 100101, China; Institute of Psychology, Chinese Academy of Sciences, CHINA

## Abstract

Self-evaluation plays an important role in adaptive functioning and is a process that is typically impaired in patients with schizophrenia. Underlying neural mechanisms for this dysfunction may be associated with manifested psychosis. However, the brain substrates underlying this deficit are not well known. The present study used brain blood oxygen level dependent (BOLD) functional magnetic resonance imaging (fMRI) and gray matter voxel-based morphometry to explore the functional and structural brain correlates of self-evaluation deficits in schizophrenia. Eighteen patients with schizophrenia and 17 healthy controls were recruited and asked to judge whether a set of personality-trait adjectives were appropriate for describing themselves, a familiar other, or whether the adjectives were of positive or negative valence. Patients had slower response times for negative trait attributions than controls did; responses to positive trait attributions were faster than those for negative traits among the patient group, while no differences were observed in the control group. Control subjects showed greater activation within the dorsal medial prefrontal cortex (dMPFC) and the anterior cingulate cortex (ACC) than the patient group during the self-evaluation > semantic positivity-evaluation contrast. Patients showed greater activation mainly within the posterior cingulate gyrus (PCC) as compared to controls for the other-evaluation > semantic positivity-evaluation contrast. Furthermore, gray matter volume was reduced in the MPFC, temporal lobe, cuneus, and the dorsal lateral prefrontal cortex (DLPFC) among the patient group when compared to controls. The present study adds to previous findings regarding self- and other-referential processing in schizophrenia, providing support for neurobiological models of self-reflection impairment.

## Introduction

Self-evaluation refers to a conscious process whereby a decision is made regarding oneself [1], or it can refer to the capacity for consciously reflecting on one’s sense of self. This process can be drawn from schemata reflecting one’s abilities, traits, and attitudes [2]. Self-evaluation is used interchangeably with terms such as “self-reflection,” “self-referential processing,” “self-awareness,” “self-appraisal, and the like. Self-evaluation is also a core part of both meta- [3] and social cognition [1, 4], which are crucial for adaptive functioning in the social environment [4].

Research suggests that capacities for self-referential processing are compromised in schizophrenia [4, 5], and self-reflection deficits may be associated with manifested psychosis [6]. More importantly, inability to self-reflect may cause impaired insight [1, 7], a common feature and important predictor of treatment adherence and prognosis in schizophrenia [8]. One recent study has shown significant relationship between self-reflection and insight in schizophrenia in brain areas related to self-reflection [8]. Additionally, compared to neurocognitive assessments or psychopathology, capacities for self-reflection are better predictors of social competence [3]. For these reasons, studies on impaired self-awareness in schizophrenia have been attracting more and more attention [9]. However, at present, the neural correlates of impaired self-reflection in schizophrenia are not well understood, and only a handful of studies have examined the neural networks underlying self-evaluation in schizophrenia.

A common paradigm for addressing self-evaluation processes in neuroscience research is to present subjects with personality-trait adjectives. Here, subjects are asked to judge whether the adjectives describe themselves or a familiar other. Functional magnetic resonance imaging (fMRI) research [1] examining normal, adult subjects has found that self-referential processing mainly relies upon the Cortical Midline Structures (CMS), which encompass the medial prefrontal cortex (MPFC), the anterior cingulate cortex (ACC), and the posterior cingulate cortex (PCC). Shad and his colleague [9] reviewed the available literature on schizophrenia and proposed a hypothetical model for deficits in self-awareness. They suggested that there is an anterior-to-posterior shift in CMS among patients with schizophrenia during self-evaluation, which was probably mediated by deficits in functional connectivity between the dorsal medial prefrontal cortex (dMPFC) and the posterior CMS. This model has been further refined based on findings from a few other studies with relatively small sample sizes [9]; however, not all studies support this model. For example, some studies [10, 11] do not observe any abnormal activation in the CMS during self-evaluation but do observe oddities in other brain areas (i.e., the temporal gyrus). Based on the aforementioned evidence, additional studies are needed to further test and refine this proposed model.

The present study utilized whole-brain fMRI, voxel-based morphometry (VBM), and structural magnetic resonance imaging (sMRI) to evaluate the functional and structural correlates of self-referential processing in schizophrenia. It was hypothesized that there would be differential neural responses during self-referential processing between patients with schizophrenia and healthy controls. These differences would be mainly observed in the CMS; these areas should also reveal differential gray matter volume between the two groups.

## Methods

### Subjects

Eighteen adult patients at Beijing HuiLongGuan Hospital in China with a DSM-IV diagnosis of schizophrenia [12] were recruited. All patients were screened to exclude for a history of major head trauma, significant medical or neurological illness, and substance abuse in the last 6 months. Patients’ current clinical symptoms were assessed with the Positive and Negative Syndrome Scale (PANSS) using 5 component scores: positive, negative, cognitive, hostility, and emotional discomfort [13]. All patients received antipsychotic medication (5 received typical neuroleptics, 12 atypical neuroleptics and medication use were missing for 1 patient.). The mean chlorpromazine equivalent dosages (CPZ) were 277.9±126.8 mg per day [14]. An additional 17 healthy controls were recruited from the surrounding community through poster advertisements. Exclusion criteria for healthy controls were (1) any mental disorders by direct psychiatric interview, (2) history of major head trauma or significant medical or neurological illness, (3) substance abuse or dependence in the past 6 months, (4) and first-degree relatives diagnosed with schizophrenia. There were no significant differences between the 2 groups with respect to age, sex, handedness, or education (Table 1). All subjects gave written informed consent, and the ethics committee at Beijing Huilongguan Hospital approved the experimental protocol. The study was conducted in accordance with the Declaration of Helsinki.

**Table 1:** Demographic and clinical characteristics of schizophrenia and controls.

Characteristics	Schizophrenia (n = 18)	Control (n = 17)	Statistics
Age(years)	40.5±5.48	41.2±3.85	*F*_1,33_ = 0.185, *p* = 0.670
Education(years)	11.1±2.70	11.3±1.26	*F*_1,33_ = 0.065, *p* = 0.801
Sex, male/female	11/7	10/7	*χ* ^2^ = 0.019, *p* = 0.890
Handedness, right/left	18/0	17/0	
Length of illness(years)^a^	15.9±6.3		
Age of onset(years) ^a^	23.4±3.6		
PANSS total scores	59.1±12.3		
Positive symptoms	8.7±3.7		
Negative symptoms	21.7±6.0		
Cognitive scores	12.1±3.3		
Hostility scores	5.8±2.2		
Emotional discomfort scores	10.8±1.6		

^a^ Date of illness duration andage of onset were missing for 1 patient.

### Self-evaluation task

The present study included three different judgment tasks, and subjects were asked to judge whether or not each personality-trait adjective was appropriate for describing the self, a familiar other (Jia Bao Wen, the premier of the People’s Republic of China from 2003 to 2012), or adjective valence (positive or negative).

Twelve lists of 9 personality-trait adjectives were selected from a pool of normalized personality-trait adjectives [15]. All adjectives consisted of two-four Chinese characters. Lists were assigned to three conditions with four lists per condition. Each list consisted of four 2-character adjectives, three 3-character adjectives, and two 4-character adjectives. Adjective valence was counterbalanced in each condition (half of the words in each condition were positive traits, the remaining half were negative traits) and was nearly counterbalanced in each list (four or five words in each list were positive traits, the remaining five or four words were negative traits).

**Fig 1:**
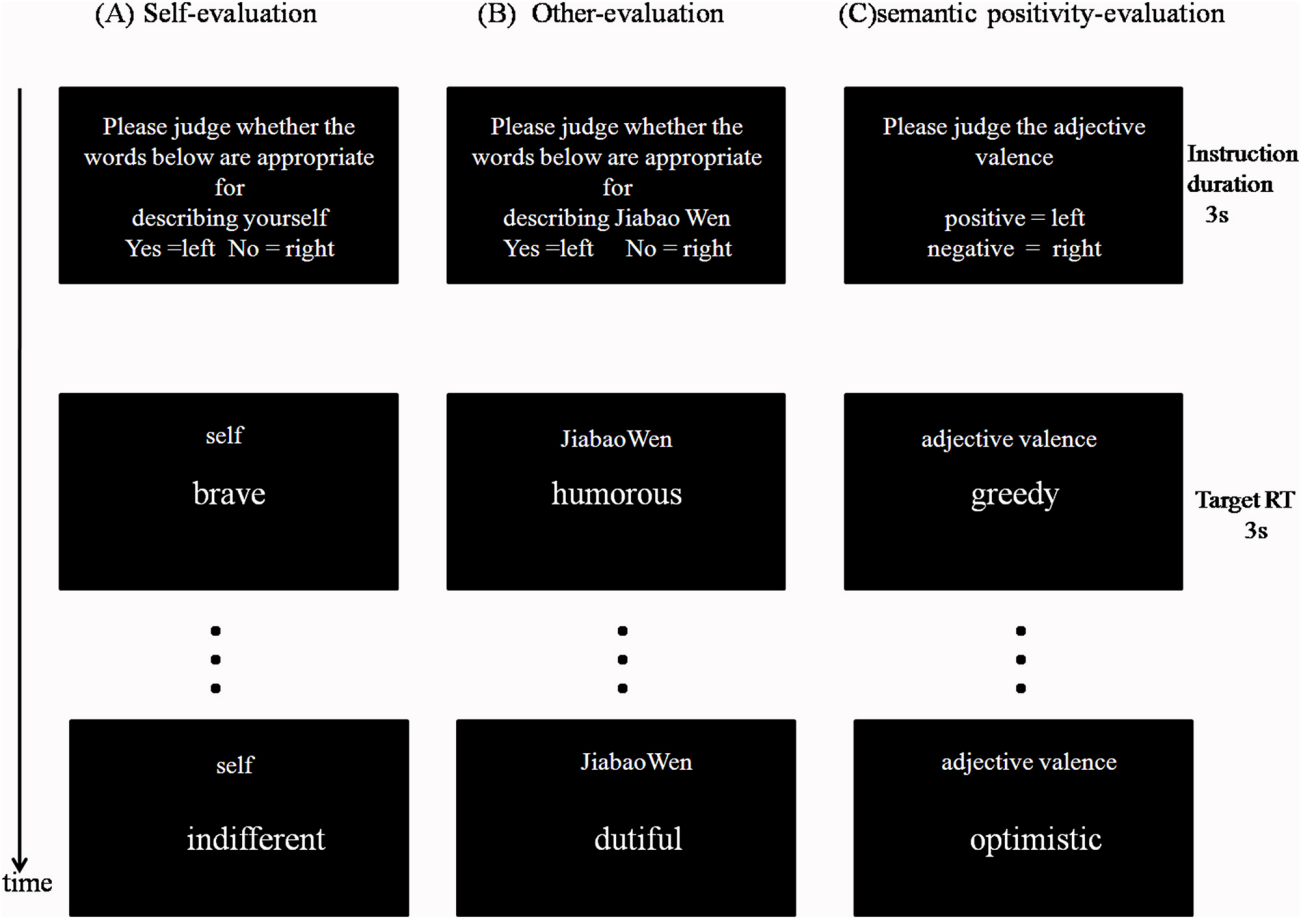
Schematic diagram of the self-reflection task. Each block started with the instruction presented in the middle of the screen (3s), followed by 9 successive pictures of personality-trait adjectives (3s each). Subjects were required to judge whether each adjective presented was appropriate for describing themselves (A), a familiar other(B), or whether the adjectives were of positive or negative valence(C).

The task consisted of one scanning run with 12 blocks. Each block was associated with one of three conditions (self-evaluation/other-evaluation/semantic positivity-evaluation), which contained 9 adjectives. Each condition was repeated 4 times, and condition order was counterbalanced as to avoid presenting the same condition during two consecutive blocks. The semantic positivity-evaluation task was used as the baseline to control for neural activation due to emotional stimulus quality, language/motoric abilities, and the task’s attentional/decision-making demands [10]. Prior to the functional run, participants were given practice trials to become familiarized with the tasks. An instruction was given before each block (e.g., “Please judge whether the words below are appropriate for describing yourself”) for 3 s, followed by 9 trials that each lasted for 3 s (Fig 1). During each trial, a small cue word (“self,” “other,” or “valence”) remained in the upper part of the screen in order to promote differentiation between the referential conditions [10] or to reduce memory demands [4]. The trial sequence in each block was randomized. Participants indicated their responses via a left- or right-handed key press mounted on a joystick. The left-/rightness of responses were counterbalanced across subjects. All text was presented in Song font, in white letters, on a black background. Visual stimuli were presented using the DMDX software package and were viewed on a back-projected screen via a head coil-mounted mirror.

### Behavioral data measures and analysis

Reaction times (RT) and responses for each of the three task conditions were recorded using DMDX. Data analyses were performed using SPSS statistics 16.0 (IBM, USA). Two 2 × 2 × 2 repeated measures ANOVAs were performed with group as a between-subjects factor and task conditions and valence as within-subjects factors to compare groups on RTs and mean proportions of self-and other-attribution.

### Image acquisition and processing

Imaging was performed on a 2-T GE/Elscint Prestige MRI scanner at Beijing 306 Hospital. Functional images were acquired during one run of 6 minutes using a gradient echo-planar image (EPI) sequence with the following parameters: repetition time (TR)/echo delay time (TE)/flip angle = 3000 ms/45 ms/90°, in-plane resolution = 2.9 mm × 2.9 mm, 6 mm slice thickness. Twenty contiguous axial slices were acquired parallel to the AC-PC line covering the whole brain with no gap. Anatomical images were acquired using a T1 weighted 3D gradient-echo sequence with TR/TE/flip angle = 25 ms/6 ms/28°, slice thickness = 2 mm with no gap, in-plane resolution = 1 mm*1 mm.

fMRI data were analyzed using Statistical Parametric Mapping software (SPM5, Wellcome Department of Cognitive Neurology, London, UK) [16]. For functional images, data were preprocessed to remove sources of noise and artifacts. Functional data were corrected for differences in acquisition time between slices for each whole-brain volume, realigned within to correct for head movement and co-registered with each subject’s anatomical data using a 6-degrees-of-freedom linear affine transformation. Functional data were then transformed into a standard anatomical space (2-mm isotropic voxels) based on the parameter obtained by normalizing its own T1 image to the ICBM 152 brain template (Montreal Neurological Institute). Normalized data were then spatially smoothed (8 mm full width half maximum [FWHM]) using a Gaussian kernel. The inclusion criteria were a maximum absolute head motion displacement of < 3 mm and rotation < 3° in x/y/z; three patients and two controls were excluded.

For the first-level fMRI analyses after preprocessing, functional images were submitted to a General Linear Model regression analysis to estimate task activation for the self-and other-evaluation conditions compared to the semantic positivity-evaluation conditions using SPM. The maps were generated using regressors convolved with a hemodynamic response function for the self-evaluation, other-evaluation, and semantic positivity-evaluation task conditions, which also included the six motion parameters as covariates of no interest. For the second-level analyses, a group analysis was performed for whole brain activity to identify general task-related activations, including self-evaluation vs. semantic positivity-evaluation, other-evaluation vs. semantic positivity-evaluation, and self-evaluation vs. other-evaluation conditions. AlphaSim was used to correct *p*-values for multiple comparisons in the whole brain to *p*-corrected < 0.01 two-tailed.

The T1 images were analyzed using the VBM5 (http://dbm.neuro.uni-jena.de/vbm.html) toolbox as part of the SPM5 software package in the following sequence: (1) A customized (i.e., a study population and tissue-type specific) template using individual T1 images from the control group was created. (2) Images were bias-corrected, tissues were classified, and then registered using linear (12-parameter affine) and non-linear transformations (warping) within a unified model20. (3) Gray matter (GM) segments were multiplied by the non-linear components derived from the normalization matrix in order to preserve actual local GM values (GM density). (4) The modulated densities were smoothed with a Gaussian kernel of 8 mm full width at half maximum (FWHM).

Two sample *t*-tests were performed on whole brain GM volume between patients with schizophrenia and controls. The group-level analysis produced differences in brain regions with significantly detectable GM volumes between the two groups. Additionally, correlation analyses were performed for associations between imaging findings and illness insight, positive symptoms and negative symptoms (PANSS) assessments in the patient group.

## Results

### Behavioral results

#### Reaction times

We excluded trials (1.96% and 9.98% of all trials, respectively, for controls and patients) where RTs were below 200 ms or above 2,500 ms. The three-way RT ANOVA revealed a significant interaction effect, *F*(1, 31) = 7.358, *p* = 0.011. Simple effects analyses observed that compared with controls (1289.21±57.48), patients had slower RTs (1,529.96 ± 59.25, *p* = 0.007) toward negative trait attributions. Moreover, for the patient group, attributions toward positive traits (1,150.39 ± 96.77) were faster than for negative traits (1529.96 ± 59.25, *p* < 0.0001), while no differences were found among the control group (*p* = 0.231).

#### Attribution style for personality traits

An analysis of attribution style revealed a significant “condition × valence” interaction effect, *F*(1, 31) = 8.01, *p* = 0.008. A simple effects analysis demonstrated that there were more positive trait attributions than negative attributions for both self- (87.0% vs. 25.9%, *p* < 0.001) and other-referential processing (90.4%vs.15.8%, *p* < 0.0001) conditions. Moreover, more negative trait attributions for the self (25.9%) were observed than negative attributions for others (15.8%), *p* < 0.0001. Additionally, there was a significant “condition × group” interaction effect, *F*(1, 31) = 4.541, *p* = 0.041. A simple effects analysis indicated that control subjects gave more “yes” answers in the self-referential condition (56.4%) than in the other-referential condition (49.9%), *p* = 0.004, while no differences were found between the two conditions among the schizophrenic group.

### Imaging results

#### Functional MRI

**Fig 2:**
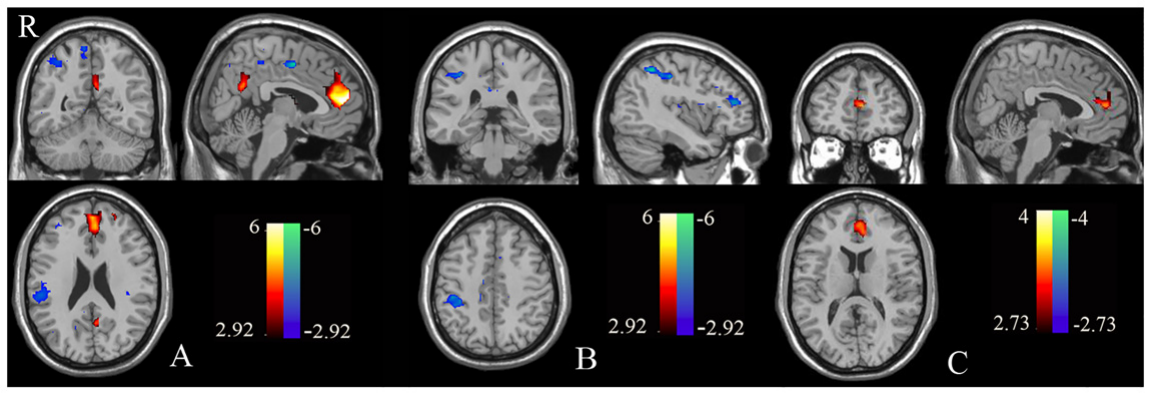
Brain regions showing self-semantic activation in patients with schizophrenia and the control group. One-sample tests produced the spatial patterns of brain regions showing self-semantic activation for both healthy controls (A) and patients with schizophrenia (B). Warm colors represent positive while cool colors indicate negative activation. A two-sample t-test assessed the differences between the two groups (controls *versus* patients, C). The above results were obtained with threshold *p* < 0.01, AlphaSim correction *p*< 0.01.

One-sample analyses indicated that the self-evaluation vs. semantic positivity-evaluation contrast revealed activation of the MPFC and PCC, as well as deactivation within the postcentral gyrus (Fig 2A) among controls and revealed deactivation within the postcentral gyrus among patients with schizophrenia (Fig 2B).

**Fig 3:**
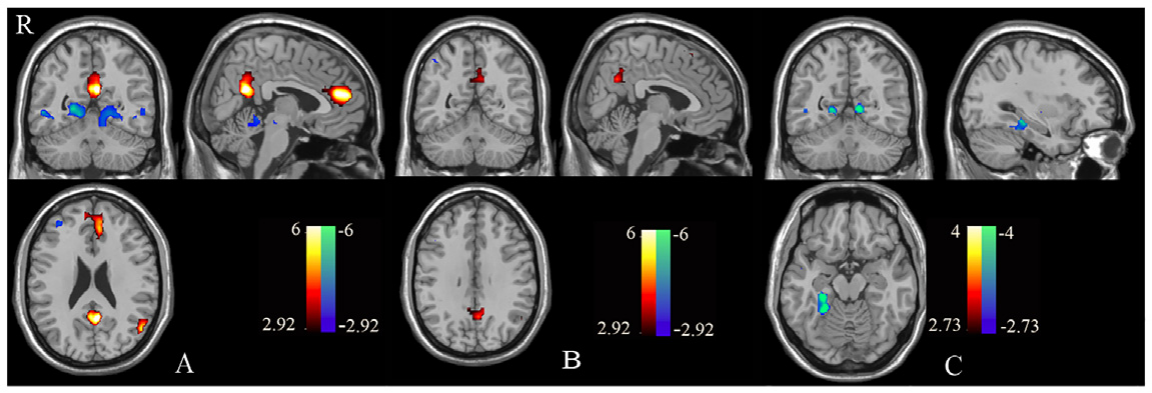
Brain regions showing other-semantic activation in patients with schizophrenia and the control group. One-sample tests produced the spatial patterns of brain regions showing other-semantic activation for both healthy controls (A) and patients with schizophrenia (B). Warm colors represent positive while cool colors indicate negative activation. A two-sample t-test assessed the differences between the two groups (controls *versus* patients, C). The above results were obtained with threshold *p*< 0.01, AlphaSim correction *p*< 0.01.

Further, among controls, the other-referential processing condition induced hyper-activation in the MPFC and PCC, as well as hypo-activation in the cerebellum relative to the semantic positivity-evaluation condition (Fig 3A); among patients, the other-evaluation vs. semantic positivity-evaluation contrast revealed hyper-activation in the precuneus and bilateral super frontal gyrus (Fig 3B).

**Fig 4:**
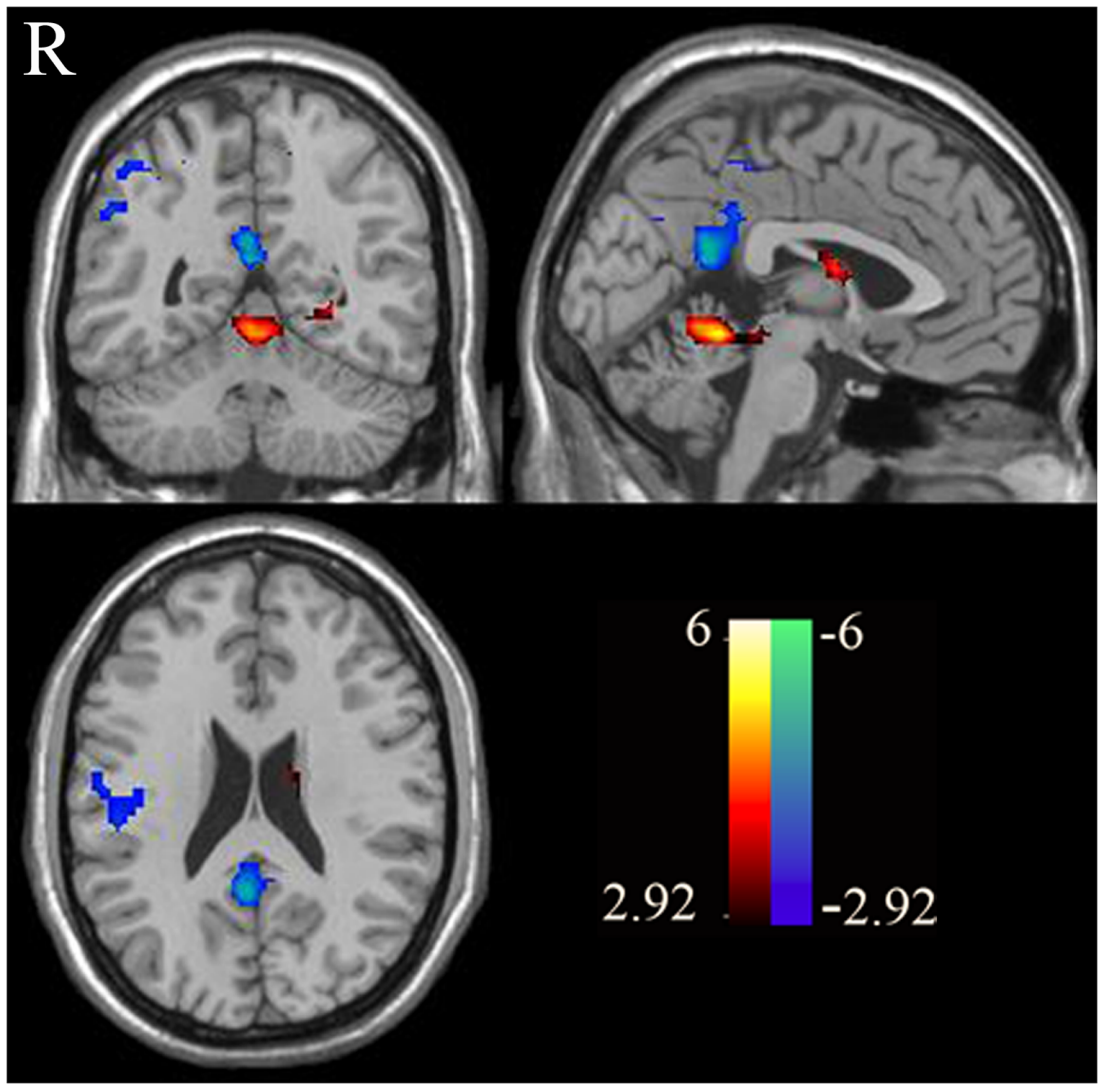
Brain regions showing self-other activation in the control group. One-sample tests produced the spatial patterns of brain regions showing self-other activation for both healthy controls. Warm colors represent positive while cool colors indicate negative activation. The above result was obtained with threshold *p* < 0.01, AlphaSim correction *p*< 0.01.

Finally, the self-evaluation vs. other-evaluation contrast revealed stronger activation in the insula and hyper-activation in the dMPFC (Fig 4) among healthy subjects, but it did not reveal any significant activation among patients.

Between-groups analysis showed that there were decreased activations of the dMPFC (i.e., dorsal areas of the anterior CMS) and the ACC in the self-evaluation vs. semantic positivity-evaluation contrast (Fig 2C and Table 2) in the patient group. Additionally, compared to controls, the patient group showed increased activation in the PCC, fusiform gyrus, and lingual gyrus in the other-semantic contrast (Fig 3C and Table 2). Finally, no significant differences were found between the two groups in the self- vs. other-referential processing contrast.

**Table 2:** Significant brain activation differences in the task between controls and patients.

Brain region	Hemisphere	BA	Cluster size	T-value	Peak voxel[x;y;z]
Self-semantic contrast					
Control>Patient					
Anterior Cingulate dMPFC	L	32	107	3.79	[-6;36;15]
Other-semantic contrast					
Control<Patient					
Fusiform Gyrus	R	37	95	5.16	[27;-33;-21]
LingualGyrus Posterior Cingulate	L	30	98	4.84	[-12;-63;0]

#### Structural MRI

**Fig 5:**
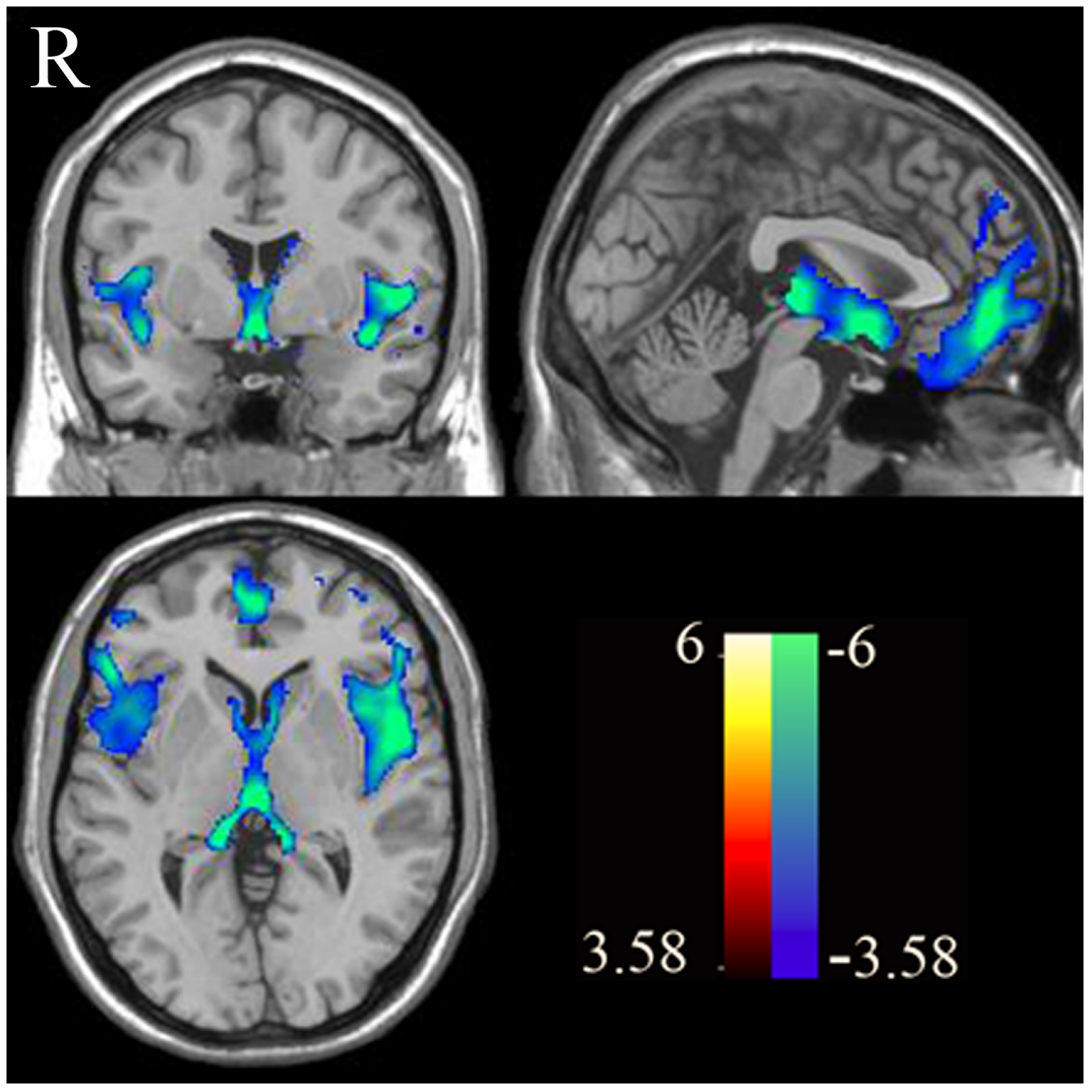
Brain regions shown significant grey volume differences between normal subjects and patients with schizophrenia. The above result was obtained with threshold *p* < 0.001, AlphaSim correction *p* < 0.01.

Compared to controls, patients with schizophrenia showed a significant gray matter volume reduction in the MPFC, temporal lobe, cuneus and the dorsal lateral prefrontal cortex (DLPFC) (Fig 5 and Table 3).

**Table 3:** Brain regions showing significant dGM differences between patients and controls.

Brain region	Hemisphere	BA	Cluster size	T-value	Peak voxel[x;y;z]
Superior temporal gyrus	L	22	9413	-8.04	[-52; 6;0]
Superior temporal gyrus	R	13	3852	-6.99	[40;6;-12]
Middle frontal gyrus DLPFC	R	10	300	-6.95	[36; 56;0]
Cuneus	R	19	156	-6.18	[32;-90;24]
Inferior temporal gyrus	L	21	455	-6.11	[-64;-10;-20]

#### Correlation analysis

No significant correlations were observed between the imaging findings and insight, positive symptoms or negative symptoms subscale from the PANSS in the patient group.

## Discussion

The present study combined fMRI and sMRI to evaluate the neural and structural correlates of self-and other-referential processing deficits among patients with schizophrenia. Additionally, a model outlining the anterior-to-posterior shift in midline cortical activity related to schizophrenia during self-reflection [9] was tested.

Behaviorally, patients had slower RTs for negative trait attributions compared to control subjects; RTs were faster for positive traits than negative traits in the patient group, while no differences were observed in the control group. These findings revealed that patients with schizophrenia need more time to process negative trait information. Additionally, there were more attributions of positive traits than negative traits in both the self- and other-referential conditions across subjects. In contrast to our study, Liu and colleagues [17] found that patients with schizophrenia were less likely and slower to endorse positive self-attributes, and more likely and quicker to endorse negative self-attributes, than control subjects. Such differences may be due to patients in the present study being inpatients with more serious negative symptoms while patients in Liu and colleagues’ study were stable outpatients with more serious positive symptoms.

At a neural level, this is one of the first studies that revealed significant hypoactivation in the dMPFC between patients and controls when self-evaluation was contrasted with non-self evaluation. To our knowledge, only one study has reported significant group differences in response to a similar contrast [18]. One of the most important limitations of that study from Bedford et al. (2012) was the small sample size, which including only 11 patients and 8 normal controls. The present study confirmed the existence of altered self-referential activity in the dMPFC with a larger sample. More importantly, the present study provided evidence regarding the role of the dMPFC in mediating self-referential activity in schizophrenia. This is in contrast to prior research [19, 20, 21] demonstrating decreased activation in the ventral medial prefrontal cortex (vMPFC), and a hypothetical model [9] of potential changes in the dMPFC during self-reflection being primarily based on data from healthy subjects. Moreover, previous studies revealed decreased activation in the vMPFC, but not the dMPFC, perhaps due to methodological differences between fMRI analytical techniques, such as an ROI-based approach [19] or a cortical surface-based analysis [20], rather than a whole brain voxel-wise analysis. It is also worth noting that even if the methods used were the same, the results would still be inconsistent. For example, using a whole brain analysis, one study [22] found group differences in the posterior CMS while in another study [18] and the present study, significant group differences were observed in the anterior CMS. This is likely due to differences in sample characteristics. Nevertheless, as the dMPFC monitors the “cognitive self” [9,23,24], decreased dMPFC activity among patients with schizophrenia in the present study may indicate that cognitively oriented decision-making, specifically when referring an object to the self, would be deficient. This may, in turn, cause inappropriate self-referential judgments and decision-making.

Beyond the dMPFC, control subjects also showed stronger activation in the ACC than patients did during self-evaluations compared to semantic positivity-evaluations. The ACC plays an important role in directing attention toward the self [1] and in emotion processing and regulation [25, 26]. Modinos et al [6] investigated the neural mechanisms underlying self-referential processing among high psychosis-proneness (PP) subjects, reporting increased activation in the ACC, insula, and dMPFC for negative self-related traits in the high PP compared to the low PP subjects. This was interpreted as attempts to diminish (e.g., activity in the dMPFC and ACC) increased emotional responses (e.g., activity in the insula) elicited by self-relevant stimuli, Perhaps the mechanisms underlying self-evaluation are different between patients with schizophrenia and high PP subjects. For instance, increased activation in the ACC and dMPFC might be a compensatory process among high PP individuals that is not present in schizophrenia.

The contrast between other-evaluations and semantic positivity- evaluations mainly demonstrated greater activation in the PCC among patients with schizophrenia as compared to healthy controls. The PCC is involved in autobiographical memory [6] and provides further information regarding past instances of self- and other-referential information [1]. This increased PCC response during other-reflections in schizophrenia may suggest that more cognitive effort is needed to draw on autobiographical memories of past situations shared with others. Several previous studies [19, 20] observed similar results to the current study, namely hyper-activation among patients with schizophrenia for other vs. baseline processing in the PCC. However, other studies [8] indicate hypoactivation in the PCC in schizophrenia for other vs. baseline contrasts. Such results may be associated with differences in baseline control conditions and experimental paradigms [8].

Finally, patients with schizophrenia showed a significant gray matter volume reduction in the MPFC, temporal lobe, cuneus and the DLPFC compared to controls, These findings confirm previous reports [27], indicating that impaired self-referential processing in schizophrenia may be related to specific structural abnormalities.

The present study has a few limitations of note. First, the patient sample included those with chronic schizophrenia who were taking atypical antipsychotic; thus, any medication effects on the present results cannot be excluded. Self-referential processing studies assessing antipsychotic-naïve patients or patients recently experiencing their first schizophrenic episode could minimize the confounding effects of long-term medication use. Moreover, similar to prior research [20], the present study did not observe significant differences in the self vs. other-evaluation contrasts. Perhaps this issue could be addressed through modifications to the chosen imaging paradigm [9]. For instance, using the patient’s name rather than a personal pronoun has been done elsewhere [20, 22]. Using trait adjectives incorporated the delusional themes of patients [22] or mental and physical illness terms [18]. Thirdly, self-reflection may be a cognitive process that underlies insight in psychosis [8]; accordingly, we performed a correlation analysis between the imaging findings and insight scores. However, no significant correlations were observed. We think that this might be because insight is a multidimensional structure [28], including illness awareness, the ability to relabel symptoms, and treatment compliance. We only used one measure of awareness in the PANSS as a dependent variable. Future studies could include scales such as the Unawareness of Mental Disorder, the Schedule for the Assessment of Insight-Extended version, and the Beck Cognitive Insight Scale to measure illness awareness and explore its relationship with self-reflection in schizophrenia. Interesting results may emerge, which will be helpful in improving our understanding of the neural correlates of impaired insight.

Despite these limitations, the present study extends previous results observing self- and other-referential processing differences in schizophrenia. The present findings revealed significant hypoactivation in the dMPFC and ACC among patients vs. controls when self-evaluation was contrasted with non-self evaluation. Furthermore, reduced gray matter volume in the MPFC among the patient group provides support for neurobiological models underlying self-reflection impairment in schizophrenia.
